# Parent Experience and Attitudes Towards Newborn Bloodspot Screening in Ireland

**DOI:** 10.3390/ijns12010002

**Published:** 2026-01-07

**Authors:** Mairéad Bracken-Scally, Anna O’Loughlin, Heather Burns

**Affiliations:** 1 National Healthy Childhood Programme, Health Service Executive, R35 TY28 Offaly, Ireland; 2 National Newborn Bloodspot Screening Laboratory, Children’s Health, D01 YC67 Dublin, Ireland

**Keywords:** newborn screening, neonatal screening, dried blood spot screening, rare diseases, congenital metabolic disorders, parent experience

## Abstract

The aim of the evaluation was to gather information on parents’ experiences and attitudes towards the Irish National Newborn Bloodspot Screening Programme (NNBSP). An interviewer-administered survey was completed by 151 parents whose babies underwent newborn bloodspot screening (NBS) between 2023 and 2025 and for whom the screening result was normal. Results suggest that NBS is highly acceptable to parents, with 100% glad their baby underwent screening. The majority (95%) felt they were provided the information needed to understand the importance of NBS for their baby, and 93% are in favour of screening for more conditions. Positive aspects of NBS reported by parents included the following: blood sampling being undertaken in the home, the sample-taker being very nice and being advised in advance to keep the baby’s heel warm to ease the sampling process. Negative aspects of NBS reported included the following: having to return to the hospital for sampling, the baby becoming distressed, not receiving adequate information and not receiving the screening results. Parents were more likely to report negative experiences if the sample was not taken at home and if the sample was taken by a healthcare professional other than a public health nurse. Parents offered recommendations for improvements to the programme. This study provides important insights into parents’ experiences and attitudes towards NBS in Ireland.

## 1. Introduction

Newborn bloodspot screening (NBS) commenced in Europe in the 1960s with the advent of programmes to screen for one or more conditions, and over time the panel of screened disorders has expanded [1]. Newborn bloodspot screening represents a robust and effective public health intervention that significantly reduces disease-specific morbidity and mortality rates when systematically maintained [2]. The National Newborn Bloodspot Screening Programme (NNBSP) was first established in the Republic of Ireland in 1966, with the implementation of screening for phenylketonuria (PKU). The programme currently offers screening for nine rare conditions (PKU, homocystinuria, classical galactosaemia, maple syrup urine disease, congenital hypothyroidism, cystic fibrosis, glutaric aciduria type 1, medium chain acyl co-A dehydrogenase deficiency and adenosine deaminase deficiency severe combined immunodeficiency), with recent (2023) ministerial approval for further expansion to include screening for severe combined immunodeficiency (SCID) and spinal muscular atrophy (SMA).

Recommendations for expansion of the Irish NNBSP are made by the National Screening Advisory Committee (NSAC), an independent committee, which advises the Minister and Department of Health on all new proposals for population-based screening programmes and revisions to existing programmes. The NSAC uses a suite of twenty criteria, based on the Wilson and Junger criteria, to appraise the viability, effectiveness and appropriateness of a proposed screening programme. These criteria are assessed through a health technology assessment, undertaken by the Health Information and Quality Authority (HIQA), and include consideration of the condition, the proposed screening method, the intervention for screen-detected individuals and implementation criteria. There should be evidence that the complete screening programme is acceptable to the target population and can be implemented. The NSAC is currently considering proposals to expand the NNBSP to screen for additional conditions, and the recently published National Rare Disease Strategy 2025–2030 [3] recommends further programme expansion.

NBS involves taking a blood sample from a newborn baby’s heel between 72 and 120 h after birth and placing the blood on a screening card. The blood sample is taken by either a midwife or a public health nurse (PHN) as part of routine postnatal care. NBS is strongly recommended but not mandatory in Ireland, and documented informed consent must be obtained from parents at the time of sample taking. To facilitate informed consent, parents receive information about NBS both antenatally and postnatally. When a baby is due to have NBS, the midwife or PHN provides the parents with verbal and written information and answers any questions they may have. Parents confirm their consent for screening by signing their baby’s bloodspot screening card. Parents are informed that, in signing the NBS card, they confirm that they received information about NBS, the information about their baby recorded on the NBS card is correct, and they consent to have their baby screened. Approximately two-thirds of NBS samples are taken in the home by PHNs during the first routine postnatal home visit. Some areas offer a weekend PHN service, while others require parents to return to the maternity hospital of birth for sample taking if this is required over a weekend.

NBS samples are sent to the National Newborn Bloodspot Laboratory (NNBSL), a single centralised laboratory where samples are analysed and results reported. Parents are not routinely informed of bloodspot screening results where no condition is suspected. In the event of a screen-positive result, parents are contacted and informed of the screening result, and clinical evaluation and diagnostic testing are arranged without delay. Each year in Ireland, approximately 130 babies with a rare condition are detected through the NNBSP [4].

Service user experience and attitudes are important aspects of service evaluation, providing information on whether services are achieving their aims, including patient-centred care, and highlighting areas for quality improvement. Additionally, information on the acceptability of bloodspot screening and attitudes to programme expansion is of value to the NSAC when considering adding new conditions to the NNBSP. In Ireland, there has been no formal evaluation of parent experience and attitudes towards the NNBSP to date, representing an important knowledge gap for the programme.

While the high uptake of the NNBSP (>99.8% of eligible babies were screened between 2020 and 2022 inclusive) [4] suggests that the programme is acceptable to parents, we cannot assume that parents would not value the opportunity to offer feedback on the programme, including highlighting areas for improvement. Additionally, the proportion of parents opting out of the NNBSP, while very low, has increased in recent years [4]. This trend is of concern to the programme, and information on parent experience and attitudes may provide valuable insights to inform interventions to address this issue. Additionally, gathering information on parent experience and attitudes is a current priority for the Irish NNBSP in the context of programme expansion to include screening for SCID and SMA, and the potential to add screening for many more conditions in the coming years.

The aim of the current project was to gain high-level insights into parent experience and attitudes towards the Irish NNBSP, inform service improvement and programme expansion and to inform future approaches to parent engagement. The scope of this evaluation was limited to the experience of parents of infants for whom the NBS results were normal/’no condition suspected’. This represents the vast majority, as conditions screened for under the NNBSP are rare, and >99% of babies screened will have normal screening results. The NNBSP is undertaking a separate targeted evaluation to explore the experience and attitudes of parents who receive a screen-positive result for their baby (<1% of all babies screened). The experience of these parents is vastly different from those who receive normal results and warrants separate, targeted consideration.

## 2. Materials and Methods

This was a service evaluation, planned and delivered in a limited timeframe by staff of the NNBSP and the NNBSL, within existing resources and in the context of multiple competing operational priorities.

A retrospective approach was adopted, and convenience sampling was used with the aim of achieving a response from a target number of 150 parents whose babies underwent NBS between December 2023 and January 2025, and for whom the screening result was normal.

The period December 2023–January 2025 was chosen as it was deemed sufficiently recent that parents would recall the experience of the bloodspot screening, but also sufficiently distant to mitigate the risk of causing distress among parents who may have had a negative experience associated with the birth of their baby or newborn screening.

Given the operational imperative to deliver this evaluation for the NNBSP in a tight timeframe and the limited resources available, a convenience sampling approach was used. Convenience sampling is a non-probability research method where participants are chosen based on ease of access and availability, rather than random selection. A clinical liaison nurse (CLN) working in the NNBSL selected NBS records at random, from babies who underwent screening in the target time period of December 2023–January 2025.

The study instrument was a survey, the design and content of which were informed by a brief review of the international scientific literature and by expert opinion. The survey was intentionally designed to be brief, taking less than five minutes to complete, with the aim of gathering key information while also respecting parent and interviewer time and maximising response rates. A ‘plain English’ approach was adopted, and cultural sensitivity was considered to ensure the language used in the survey was accessible and appropriate to the target audience.

The survey included questions pertaining to the overall experience of bloodspot screening, attitudes towards expansion of the programme, whether information received in advance of screening was sufficient to understand the importance of this service, and any particularly positive or negative experiences parents may recall. Parents were also invited to provide recommendations for improvements to the screening programme. Response options were primarily binary (‘yes’/‘no’), with free-text boxes provided for the optional inclusion of further information.

Key demographic data, routinely collected by the NNBSP on the bloodspot screening card at the time of sample taking (date of NBS, Eircode and ethnicity), were sourced from programme records. Principles of data minimisation were followed, with demographic variables chosen to allow stratification of results by key factors such as ethnicity and area-level deprivation. Parents were also asked about where the bloodspot sample was taken (primarily maternity hospital or community) and by whom (primarily midwife or public health nurse), with the aim of identifying any variation in parent experience by these factors. The survey was inputted into SmartSurvey and all data were collected via this platform.

The survey was interviewer-administered over the phone by a CLN working in the NNBSL. This approach enabled the interviewer to ensure that parents understood that the survey pertained to NBS and to clarify any queries that arose. Data were collected over a 13-week period from March to June 2025, with phone calls being made at times that were convenient for the CLN, noting the clinical imperative to prioritise day-to-day service provision. Having been provided with participant information and offered the opportunity to seek further information, parents were asked to provide their consent to complete the survey. Parents were made aware that they could cease the process and end the call at any stage. As the survey was fully interviewer-administered, this allowed the interviewer to clarify any issues that arose at the time of data collection and provide additional information where required.

Descriptive and inferential statistical analyses were undertaken, with chi-square tests used to explore relationships between categorical variables.

As this project represented a routine service evaluation, a Research Ethics Committee review was not required. However, ethics were a key consideration for the project group, and ethical principles were followed at all times.

## 3. Results

Of a total of 188 parents contacted, 86% (162/188) consented to participate in the survey. Of these, 7% (11/162) did not recall the experience of NBS and so were unable to participate further. The complete sample size for the survey was therefore 151. Data were complete for some, but not all, questions and demographic variables. All data collected were included in the analyses.

### 3.1. Respondent Demographics

**Figure 1:**
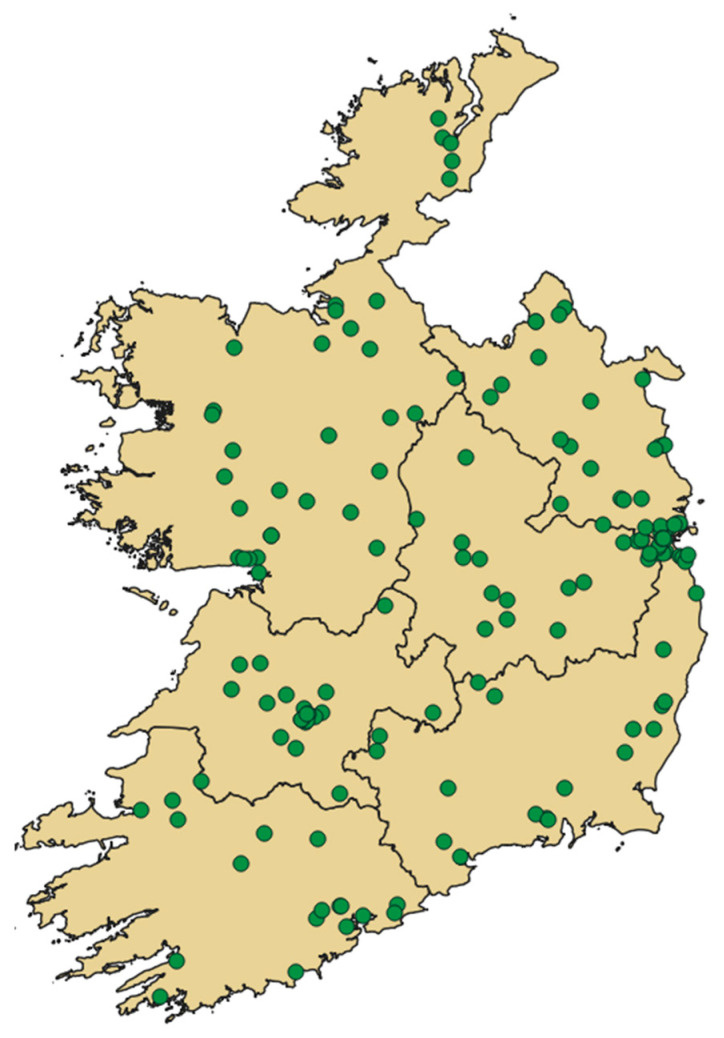
Mapping of survey respondents by health regions of HSE.

An overview of respondent demographics is presented in Table 1. The majority of surveys (99%) were completed by the mother of the screened baby. The ethnicity of respondents broadly aligns with that of the overall population of Ireland, with census data from 2022 reporting that 82% of the population identified as White Irish, 9% as “Any other white background” and 0.7% as Irish Travellers [5]. Approximately 41% of respondents lived in areas of greater socioeconomic deprivation, while 59% lived in areas that were marginally above average (45%) or affluent (14%), broadly similar to the general Irish population [6]. There was broad geographical representation, with responses relatively evenly spread across the six health regions of the HSE, and some expected concentration noted in Dublin, the capital city, where approximately 25% of the population resides (Figure 1).

Proportions of samples taken by PHNs in the community/at home (68%) versus by the midwifery service in a hospital setting (~30%) broadly align with information held by the NNBSP regarding the location of sample taking.

### 3.2. Experience and Attitudes Towards NBS and Expansion of NNBSP

**Figure 2:**
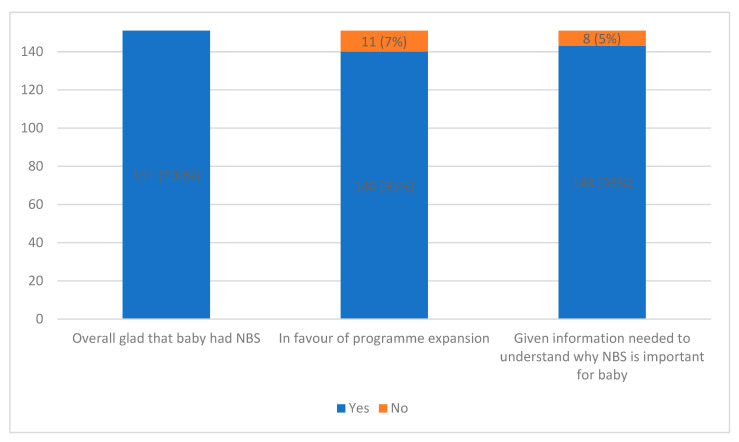
Experience and attitudes towards NNBSP and expansion of NNBSP.

All respondents (100%, *n* = 151) were glad that their baby underwent NBS (Figure 2). Two parents provided further free-text information, noting positive aspects such as the “peace of mind” and “sense of relief” provided by NBS.

The majority (93%, *n* = 140) of parents indicated that they would be in favour of the programme screening for more conditions (Figure 2). Thirty-four parents (22.5% of respondents) provided further free-text information, with six specifically noting that they would be in favour of expansion “once no more blood is needed from the baby”. The evaluation sample size was not sufficiently powered to allow stratification of reporting on whether respondents were in favour of programme expansion by demographic variables.

The majority of parents (95%, *n* = 143) reported being given the information they needed to understand why NBS was important for their baby (Figure 2). Fourteen parents (9%) provided additional free-text information, with four noting that they did not receive detailed information about the test and/or did not know what conditions were being screened for. For example, one parent outlined: “I just did it because all my family and friends had it done for their babies but I don’t even know what conditions are included in the programme.”

#### 3.2.1. Positive Aspects of Parent Experience of NBS

Of the 151 parents surveyed, 66 (44%) provided information on positive aspects of their experience of newborn screening, including: having the test undertaken at home (*n* = 25); the sample-taker being very nice (*n* = 22); the test being conducted quickly (*n* = 12); being advised in advance to keep baby’s heel warm (*n* = 7); and being allowed to leave the room to avoid seeing baby distressed (*n* = 2). A selection of illustrative quotations is provided below.

*“It was really great that I didn’t have to leave the house for the test!”*

*“The public health nurse was very kind. It was very convenient to have the test done in our home and not to have to leave the house with a newborn.”*

*“The PHN telling us in advance to have baby’s heel warm helped as she was able to get the sample really quickly and got enough blood to fill the card!”*

*“The midwife let me leave the room while she was taking it as I didn’t want to see.”*

Likelihood of reporting on positive aspects of the experience of newborn screening did not differ significantly based on where the sample was taken (i.e., at home or otherwise), who took the sample (i.e., PHN or otherwise), ethnicity of the parent (i.e., White Irish or otherwise) or area-level deprivation (above/below the average level of deprivation, disadvantaged or affluent).

#### 3.2.2. Negative Aspects of Parent Experience of NBS

Of the 151 parents surveyed, 10 (7%) provided information on negative aspects of their experience of newborn screening, including: having to return to the maternity hospital for the test following discharge (*n* = 4); baby becoming distressed (*n* = 3); not receiving adequate information (*n* = 2); and not receiving screening results (*n* = 2). A selection of illustrative quotations is provided below.

*“Having to come back into maternity hospital the day after discharge for the test post c-section with two other children at home.”*

*“It took a while for the nurse to get the blood from the baby which was quite distressing.”*

*“Not getting a leaflet with information before the test. Not getting results back and having to assume they’re all okay.”*

Reporting of negative experiences was significantly more common in parents where the sample was taken in a healthcare setting (18%, 9/49), compared to at home (3%, 3/102); χ^2^ = 10.77, *p* = 0.001. Relatedly, reporting of negative experiences was significantly more common in parents where the sample was taken by a nurse or a midwife (19%, 9/48) compared to a PHN (3%, 3/103); χ^2^ = 11.23, *p* < 0.001. There were no significant differences in the likelihood/prevalence of reporting negative experiences based on ethnicity or area-level deprivation.

#### 3.2.3. Suggestions for Improvement to the NNBSP

Of the 151 parents surveyed, 22 (15%) provided suggestions for improvements to the NNBSP, including recommending that screening results are routinely communicated to parents (*n* = 8); provision of improved information at time of sampling (*n* = 7); provision of a weekend/bank holiday PHN sample-taking service (*n* = 4); being advised in advance to warm the baby’s heel (*n* = 2); and provision of information in the parent’s first language (*n* = 1). A selection of illustrative quotations is provided below.

*“It would be nice to get the results issued to parents just to be 100% sure everything is okay.”*

*“To give parents results even if they’re normal for peace of mind for parents.”*

*“Better information at time of sampling as I didn’t remember the antenatal information.”*

*“PHNs being available on weekend days to do the screen to save parents travelling back into maternity hospitals.”*

*“Telling parents in advance to keep baby’s heels warm so test is more likely to work the first time.”*

Likelihood of providing suggestions for improvements to the NNBSP did not differ significantly based on where the sample was taken, who took the sample, ethnicity of the parent or area-level deprivation.

## 4. Discussion

This evaluation provides valuable insights into parents’ experiences and attitudes towards NBS in Ireland. The findings support inferences previously drawn from the high uptake rates of NBS that the programme is acceptable to, and valued by, parents and guardians, with 100% of parents surveyed indicating that they are glad their baby underwent screening. This aligns with international findings, with a 2025 systematic review demonstrating consensus support for NBS among parents [7]. Additionally, recent (2022) research in the United States demonstrated that the majority (77%) of parents whose child(ren) received normal screening results agreed that newborn screening for infant-onset conditions is “a positive thing”, and more than three quarters (75.4%) agreed with the statement “I am glad my child had newborn screening” [8]. Similarly, an American study published in 2022 reported that 96% of parents believed that every newborn should receive standard newborn screening [9].

The majority of parents (95%) reported being provided the information they needed to understand why NBS was important for their baby, suggesting that the programme is striking the right balance in providing enough information to support informed consent, without overwhelming parents, for the majority of whom screening results will indicate that no condition is suspected for their baby. However, some parents explicitly recommend provision of more information at the time of sample-taking, noting challenges in recalling information provided antenatally. This highlights the importance of providing parent information at multiple junctures (including antenatally and at the time of sample-taking), in multiple formats and in a range of languages, including practical advice in advance of sample taking on the importance of keeping the heel warm.

As described in the introduction, the Irish NNBSP is expanding to include screening for SCID and SMA, and the NSAC is considering proposals for the addition of further conditions. The NSAC considers multiple criteria pertaining to the viability, effectiveness and appropriateness of screening to inform its recommendations. These criteria include the acceptability of screening to the target population. This evaluation offers relevant information attesting to broad parental support for expansion of the NNBSP, with 93% indicating that they would be in favour of the programme screening for more conditions. This is in line with international findings, with a recent (2025) systematic review reporting consensus support among parents for expanding NBS to include additional conditions [7]. In the Netherlands, van der Pal et al. [10] found that 95% of parents who had participated in newborn screening were positive about expansion of the programme, while van Dijk et al. [11] reported that both parents and healthcare professionals favoured screening for more conditions, with the caveat that screening should focus on early detection of conditions for which effective treatment is available (a core tenet of population-based screening). In the Irish evaluation, a number of parents explicitly noted support for programme expansion as long as no further sampling is required from the baby. This highlights the importance of high-quality sample-taking and minimising avoidable repeat sampling. Negative experiences of NBS cited by parents in Ireland included the distress and discomfort caused to the infant. Evidence attests that warming the baby’s heel in advance of NBS sample-taking results in lower pain scores and a significantly shorter sampling time [12], as well as a shorter duration of crying [13]. In the current evaluation, a number of parents specifically commented on the value of receiving practical advice on warming the baby’s heel in advance of sample-taking. This again highlights the importance of providing practical advice to parents in a timely manner to support high-quality sample taking that is as quick and painless as possible.

Research has shown that attitudes towards screening are significantly impacted by relationships with key healthcare professionals [14]. The findings of this evaluation support this assertion, with reporting of negative experiences significantly more common in parents where the sample was taken in hospital by a nurse or midwife, compared to instances where the sample was taken by a PHN, who typically takes the sample in the home environment and establishes a relationship with the parents through a series of visits in the postnatal period. In the Irish context, ensuring national provision of weekend PHN sampling services could serve to improve the parent experience of NBS. The evaluation provides reassuring parent feedback on positive aspects of the Irish NNBSP, such as the ability to have the sample taken within the home and the kindness of sample takers. Key areas for potential improvement have also been highlighted, including the provision of screening results to all parents, the provision of improved information at the time of sampling, the provision of a weekend/bank holiday PHN sampling service, consistently advising all parents to warm the baby’s heel in advance of sample-taking and the provision of information in the parents’ first language.

Strengths of this evaluation include the fact that it provides, for the first time in Ireland, key insights into parents’ attitudes and experiences towards NBS. The high response rate attests to the effectiveness of the interviewer-administered approach and the acceptability of the survey to the target population. The interviewer-administered nature of the survey meant the interviewer (CLN) could ensure that parents understood survey questions and context, and could clarify any queries that arose during data collection.

A key limitation of the evaluation is the convenience sampling approach used to recruit participants. The evaluation was delivered by staff of the NNBSP and NNBSL, within existing resources, to a tight deadline and in the context of multiple competing operational priorities. Convenience sampling was an appropriate approach in this context, as it represents a practical, pragmatic approach, often used when time and resources are limited. However, findings cannot be considered representative of the target population, and further research is recommended to produce generalisable findings. Despite this limitation, the evaluation met its intended aim of gathering high-level insights into parent experience and attitudes towards the Irish NNBSP, to inform the ongoing delivery, expansion and quality improvement in this essential service.

## Figures and Tables

**Table 1:** Key demographics of respondents.

	***n***	**%**
**Survey completed by (*n* = 151)**		
Mother	150	99%
Father	1	<1%
**Ethnicity (*n* = 147)**		
White Irish	110	75%
White Irish Traveller	2	1%
Any other White background	11	7%
Black or Black Irish—African	2	1%
Black or Black Irish—any other Black background	1	<1%
Asian or Asian Irish—Chinese	4	3%
Asian or Asian Irish—any other Asian background	12	8%
Other ethnicity, including mixed background	5	3%
**Area-level deprivation (as per parents’ home address)** ^1^ **(*n* = 149)**		
Very disadvantaged	4	3%
Disadvantaged	16	11%
Marginally below average	40	27%
Marginally above average	67	45%
Affluent	21	14%
Very affluent	1	<1%
**Location of NBS (*n* = 151)**		
At home	102	68%
In a maternity hospital	47	31%
In a children’s hospital	1	<1%
In local health centre	1	<1%
**Sample taken by (*n* = 151)**		
Public Health Nurse	103	68%
Midwife	43	28%
Hospital nurse	5	3%

^1^ Based on the Pobal HP Deprivation Indices. This index shows the level of overall affluence and deprivation of areas and is updated for each national census.

## Data Availability

Data are unavailable due to privacy and ethical restrictions.

## Abbreviations

The following abbreviations are used in this manuscript:

CHI	Children’s Health Ireland
CLN	Clinical Liaison Nurse
HIQA	Health Information and Quality Authority
NBS	Newborn bloodspot screening
NCIMD	National Centre for Inherited Metabolic Disorders
NNBSL	National Newborn Bloodspot Screening Laboratory
NNBSP	National Newborn Bloodspot Screening Programme
PHN	Public Health Nurse
PKU	Phenylketonuria
SCID	Severe Combined Immunodeficiency
SMA	Spinal Muscular Atrophy
